# Role of NMR in High Ordered Structure Characterization of Monoclonal Antibodies

**DOI:** 10.3390/ijms22010046

**Published:** 2020-12-22

**Authors:** Yuji Tokunaga, Koh Takeuchi

**Affiliations:** 1 Molecular Profiling Research Center for Drug Discovery, National Institute of Advanced Industrial Science and Technology, Tokyo 135-0064, Japan; tokunaga.y@aist.go.jp; 2 Cellular and Molecular Biotechnology Research Institute, National Institute of Advanced Industrial Science and Technology, Tokyo 135-0064, Japan

**Keywords:** NMR, high ordered structure, monoclonal antibodies, multi domain, formulation condition, ^15^N-direct detection

## Abstract

Obtaining high ordered structure (HOS) information is of importance to guarantee the efficacy and safety of monoclonal antibodies (mAbs) in clinical application. Assessment of HOS should ideally be performed in a non-invasive manner under their formulated storage conditions, as any perturbation can introduce unexpected detritions. However, most of the currently available techniques only indirectly report HOS of mAbs and/or require a certain condition to conduct the analyses. Besides, the flexible multidomain architecture of mAbs has hampered atomic-resolution structural analyses using X-ray crystallography and cryo-electron microscopy. In contrast, the ability of nuclear magnetic resonance (NMR) spectroscopy to structurally analyze biomolecules in various conditions in a non-invasive and quantitative manner is suitable to meet the needs. However, the application of NMR to mAbs is not straightforward due to the high molecular weight of the system. In this review, we will discuss how NMR techniques have been applied to HOS analysis of mAbs, along with the recent advances of the novel ^15^N direct detection NMR strategy that allows for obtaining the structural fingerprint of mAbs at lower temperatures under multiple formulation conditions. The potential application of these NMR strategies will benefit next-generation mAbs, such as antibody-drug conjugates and bispecific antibodies.

## 1. Introduction

Advancements in biotechnology have expanded the pharmacological modalities beyond the conventional small molecules. The expansion provides an opportunity to establish a novel strategy to target diseases, even for those which have been difficult to cure. Among such new modalities, biologics, especially therapeutic monoclonal antibodies (mAbs), have been utilized more extensively in clinics over the last decade. There is a nearly three-fold increase in the number of therapeutic mAbs on the market in 2019, as compared to 2010, and more than 600 mAbs are currently in commercial clinical pipelines [1].

**Figure 1:**
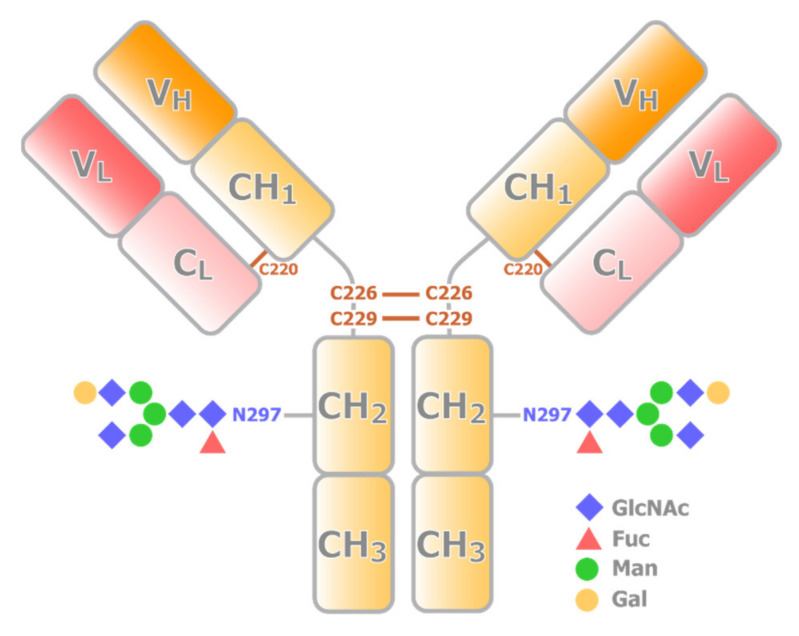
Schematic representation of the structure of IgG. An IgG consists of two heavy chains (yellow) and two light chains (red). The V_H_ and CH_1_ domains from a heavy chain interact with a light chain to form a Fab portion, while CH_2_ and CH_3_ domain dimer forms Fc portion. Fab and Fc portions are connected by the hinge region, where multiple conserved disulfide bridges are present. CH_2_ domain has a conserved N-glycosylation site.

Most therapeutic mAbs are IgG1, representing the most abundant and most extensively studied subclass of human IgG. IgG1 is a tetrameric protein consisting of two heavy chains with V_H_, CH_1_, CH_2_, and CH_3_ domains, and two light chains with V_L_ and C_L_ domains. Structurally, V_H_, CH_1_, V_L_, and C_L_ domains together form a Fab portion (Figure 1). On the other hand, two CH_2_ and two CH_3_ domains form an Fc portion. Thus, IgG1 also has dynamic nature as a multidomain protein. Fab is responsible for specific high-affinity binding to antigens, whereas the Fc region mediates immune response and effector functions. Fab and Fc fragments are connected by a hinge region, which is composed of 23 a.a. residues (216-EPKSCDKTHTCPPCPAPELLGGP-238). The two heavy chains are connected by two disulfide bridges, Cys-226 and Cys-229, at the hinge. A disulfide bridge at Cys-220 connects the light chain and heavy chain. The former half of the hinge provides flexibility to Fab, allowing IgG1 to bind to multiple antigens simultaneously. Currently, advances in protein engineering allow for constructing bispecific antibodies, which can target two different epitopes using different Fabs in a single IgG [2]. Taking advantage of the multivalent nature, bispecific antibodies have been extensively utilized in clinical applications.

The Fc portion, in contrast, is indispensable for antibody effector functions, such as antibody-dependent cell-mediated cytotoxicity (ADCC), antibody-dependent cellular phagocytosis (ADCP), and complement-dependent cytotoxicity (CDC), through Fc-FcγR and Fc-Complement 1q (C1q) interactions [3]. The Fc portion is basically composed of two identical CH_2_ and CH_3_ domains, which are related to each other by a two-fold axis. Thus, each Fc portion has been considered to bind up to two FcγR molecules. Indeed, two X-ray crystal structures suggested the 1:2 stoichiometry interactions, albeit the proposed models of the IgG-FcγR interactions are controversial [4,5]. Structural characterization by solution NMR, along with other biophysical methods, however, revealed that the stoichiometry of the interaction is 1:1 in reality. The binding of FcγR onto one of the two symmetrically related sites on the Fc region induces a conformational change in the other site to prevent the binding of another FcγR [6]. This fact clearly represents the importance of characterizing the interaction and structure of mAbs in physiological solutions.

High ordered structures (HOSs) of mAbs are critical in clinical applications, as these are directly reflected in their bioactivity [7]. The HOS of the biologics can be compromised by partial denaturation, aggregation, and chemical alterations. Tertiary structures of the biologics can be affected by the difference in expression, purification, formulation, and storage conditions, and the undesired HOS of mAbs often reduces their efficacy and causes unexpected adverse effects. Therefore, biophysical assays to analyze their HOS should be performed throughout the research and development phases to avoid such issues. In such a circumstance, assessing the HOS under their formulated storage conditions is essential to ensure the bioactivity and safety of a mAb. Such an analysis should ideally be performed in a non-invasive manner, as any perturbation of the sample can introduce undesired or unexpected defects to the system.

In addition, mAb has posttranslational modifications (PTMs), such as glycosylation. Each of the CH_2_ domains possesses one N-linked oligosaccharide site at Asn-297 (Figure 1), and some also have glycosylation at Fab. The compositions of sugar chains are sensitive to production conditions and can modulate the stability, bioactivity, and immunogenicity of the biologics [8,9]. Thus, quantitative analysis of the PTM states of the intact biologics is also important in structural evaluation. The Fc glycosylation of IgG is also critical to antibody effector functions [3]. Optimization of Fc glycosylation has been explored to enhance the ADCC activity of therapeutic mAbs, and a high degree of galactosylation can promote activation of CDC and have modest effects on ADCC [10,11]. It is also known that decreases in fucose levels lead to an increased affinity of IgG1 to FcγR, and an enhancement of ADCC activity [12]. The glycoform-dependent conformational changes of the Fc portion of IgG have been extensively analyzed by solution NMR [13]. It has been shown that the trimming of the carbohydrate residues results in the structural perturbation in the CH_2_ domains as well as a part of the lower hinge region, including the FcγR-binding site, where there is no direct contact with the sugar chain. These results indicate that the sugar chain is an integrated part of the IgG structure and is required for maintaining the structural integrity of the FcγR-binding site. It should be noted that, with sophisticated stable isotope labeling, NMR has been contributing to direct analysis of the structure and dynamics of sugar chains in IgG [14,15].

To characterize HOS of mAbs, biochemical and biophysical strategies, such as H/D-exchange mass spectrometry, differential scanning calorimetry, size exclusion chromatography, light scattering, and scanning electron-assisted dielectric microscopy, etc., have been utilized [16,17,18]. Several spectroscopic strategies, such as near- and far-UV circular dichroism, Raman, and Fourier-transform infrared, have also been used to evaluate the tertiary structures of the biologics [19,20,21]. However, these techniques often only indirectly report HOS of mAbs and require a certain condition to conduct these analyses. Although atomic-resolution structures of the biologics can be obtained by X-ray crystallography or cryo-electron microscopy, the requirement for crystallization or freezing procedures potentially modulates their tertiary structures. In addition, due to the flexible domain arrangements in intact mAb, only limited atomic-resolution structural information is available for full-length mAbs, and these structures can be only a single snapshot of the multiple conformations that exist in solution [22]. Therefore, a strategy that allows a non-invasive evaluation of the tertiary structures of the biologics is needed and, especially, those able to detect rarely populated structural states that can cause deterioration or side effects.

Nuclear magnetic resonance (NMR) spectroscopy offers a unique opportunity to non-invasively obtain structural information of the biologics under their formulated conditions, as it permits the structural analysis of biomolecules in various solutions [23,24,25,26,27,28,29,30,31,32]. As has already been discussed in the interaction between the Fc portion of IgG and FcγR, it is of importance to characterize the proteins in solution to gain a correct view of the systems. In this review, we will discuss how NMR has been utilized in biophysical and structural characterization of mAbs, along with the recent advances of the novel ^15^N direct detection NMR strategy that allows one to obtain the structural fingerprint of intact mAbs at a wide range of temperatures and formulations.

## 2. Use of ^1^H-Detected 1-Dimensional NMR to Characterize mAbs

Since the chemical shifts and line shapes of proton resonances are sensitive to the structure and dynamics of each position, the simplest ^1^H-detected 1-dimensional (^1^H-1D) NMR spectrum should reflect changes in HOS of a mAb. However, the application of ^1^H-1D NMR to a formulated mAb is not straightforward. Since most of the formulated conditions contain additives such as sugar, amino acids, and surfactants, the NMR spectra are overlapped with much stronger signals from these formulation components. In addition, severe line broadenings due to large molecular weight of mAb impair characteristics in the ^1^H NMR spectrum and small changes in HOS can be masked by the major of unaffected part of the mAb. Thus, additional tricks are usually applied to enhance the difference between mAbs. For example, in the PROtein FIngerprint by Line shape Enhancement (PROFILE) method [33], differences in the translational diffusion rates between large antibodies and small formulation components are exploited to distillate a ^1^H-1D spectrum of the mAb. Then, the featureless component in the spectrum is removed by subtracting the artificially broadened spectrum to yield a PROFILE spectrum, which mostly contains unique components in the mAb spectrum. It is shown that the spectral correlation analysis of the PROFILE spectra can discriminate a pair of mAbs with different sequences and, even, the same mAb in different formulations [33]. Besides, it has been proposed that efficient spin-diffusion among protons in the protein’s structured regions can be utilized for selective detection of HOS components in the mAb NMR spectra [34].

^1^H-1D NMR is also useful in monitoring the reversible self-association of mAbs, which can be the first step toward irreversible events, such as aggregation and liquid–liquid phase separation [35,36]. It has been shown that the mAb signal intensity in ^1^H-1D NMR spectra reflects the size of transient protein clusters and/or overall solution viscosity [35,36]. When two different mAbs, rituximab and infliximab, are compared, the infliximab gave lower S/N, which might be due to its tendency to form reversible oligomers [37]. In addition, translational diffusion coefficients and transverse relaxation rates can be measured to analyze these factors more quantitatively.

It should be noted that ^1^H-detected 1D NMR experiments are also used to analyze the interaction between formulation components and biologics [38]. Finding formulations to provide sufficient stability during long-term storage is important in developing a therapeutic mAb. For this purpose, saturation transfer difference NMR (STD-NMR) spectroscopy was used to assess the interactions of formulation components with mAb to understand the mechanism of stability enhancements by these additives [38].

## 3. Use of Heteronuclear-Detected 1D NMR for Structural Characterization of mAb

The heteronuclear-detected 1D NMR of a mAb has a long-standing history. Already in the late 1980s, Arata and co-workers have shown that specific detection and assignment of mAb signals is possible for intact IgGs, using carbonyl ^13^C direct-detection NMR spectroscopy [39], along with double-labeling techniques [40,41]. By growing mammalian cells in a serum-free medium, the authors have successfully introduced [1-^13^C] Met into a mouse mAb and established the complete assignment of Met carbonyl ^13^C resonances. The strategy was expanded to the carbonyl carbons of Trp, Tyr, His, and Cys residues, and used for the structural analyses of antigen binding, domain–domain interactions [42], and dynamics of the hinge region [43]. These strategies are fundamental to the multinuclear approach of mAb NMR spectroscopy [44].

Large self-associated species of mAb are no longer visible in a conventional NMR spectrum. However, such an NMR-invisible state can be accessible by using the dark-state exchange saturation transfer (DEST) NMR techniques [45,46]. In the DEST experiment, the saturation of the invisible “dark” state is transferred to the NMR visible state by an exchange between visible and invisible states, leading to signal attenuation. Using two differentially ^19^F-labeled mAbs [47], the temperature-dependent formation of protein-specific clusters was assessed at high concentrations (up to 400 mg/mL) [48]. The strategy would be useful in studying mAb mixtures to see if there are any reversible aggregation/oligomerization that are not readily detected in other experimental strategies.

## 4. Obtaining Structural Fingerprint by ^1^H-Detected Multidimensional NMR

The 1D experiments are more sensitive in general; however, site-specific detection of resonances is not straightforward, without introducing the additional trick such as specific labeling and/or extensive post-acquisition processing. In contrast, multidimensional NMR spectra can offer a comprehensive fingerprint of a protein, in which most of the sites are individually resolved in the spectrum. Especially, the application of two-dimensional (2D) heteronuclear ^1^H,^15^N- or ^1^H,^13^C-correlated spectroscopy to the characterization of HOS of mAb has generated great interest.

However, the application of multidimensional NMR methods to intact mAbs is a significant challenge due to short transverse relaxation times due to slow molecular tumbling. For such large molecular weight systems, the deuteration of nonlabile ^1^H sites and use of transverse relaxation optimized spectroscopy (TROSY) or cross-correlated relaxation-enhanced polarization transfer (CRINEPT) are commonly employed to overcome the rapid transverse relaxation [49,50]. Although heterologous expression of mAbs in *Escherichia coli* is possible to achieve high-level deuteration, mAbs obtained in this approach do not retain mammalian-type glycosylation. In contrast, using mammalian cell lines, appropriately glycosylated mAbs can be produced [15]; however, high-level deuteration is difficult as these cells cannot grow in such conditions.

**Figure 2:**
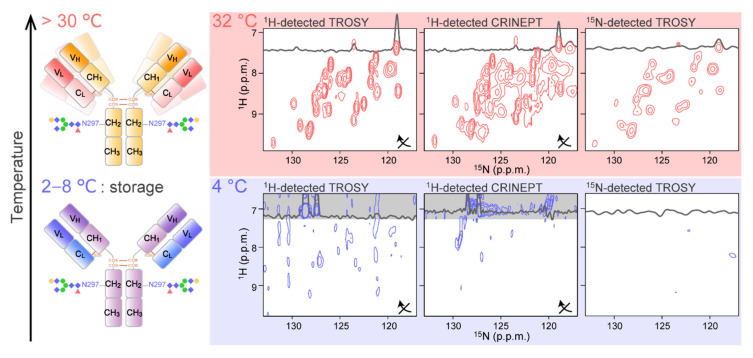
Application of conventional 2D ^1^H-^15^N correlation experiments to mAb. The spectra were recorded at (top) 32 °C and (bottom) 4 °C for an intact [^15^N-Ala] IgG. For each condition, the ^1^H-detected TROSY (left), ^1^H-detected CRINEPT (middle), and the ^15^N-detected TROSY (right) were recorded. For ^1^H-detected spectra, the ^1^H and ^15^N axes of the spectra are transposed as indicated by arrows. The figure is reproduced from [67].

The general strategy to obtain the 2D NMR spectra of mAb is to use high temperatures to accelerate the molecular tumbling (Figure 2; upper). In addition, fragmentation of intact IgG into Fab and Fc portions is also widely used to reduce the molecular weight. Indeed, almost complete assignment of the Fc region is reported in such a condition [51]. Currently, high-temperature recording at natural isotopic abundance is possible for both ^13^C and ^15^N [52], with the implementation of high-sensitive cryogenic probe technology [53] and the utilization of rapid acquisition methods, including selective optimized flip angle short transient (SOFAST) pulse schemes [54] and the non-uniform sampling (NUS) [55,56]. It is reported that more than 95% of mAb ^1^H^13^C methyl resonances can be observed in a spectrum acquired in 12 h at 900 MHz and 50 °C [52]. The sample concentration was limited to be <300 μM due to increases in sample viscosity at higher concentrations. At 600 MHz, a 2D ^1^H^13^C NMR methyl fingerprint spectrum was also be acquired; however, a reduction in the spectral resolution was reported. As for 2D ^1^H^15^N correlation spectra, a SOFAST–heteronuclear multiple quantum correlation (HMQC) spectrum of NISTmAb was obtained with a total experimental duration of approximately 68 h at 900MHz and 50 °C [57]. Such spectra have been shown to have the ability to distinguish the different mAbs [57], different glycosylation states [57,58], the effect of additives [59], and chemical modifications [60,61]. In most cases, however, Fab and Fc fragments were used for detailed analyses [62]. It was reported that Fc and Fab fragments yielded methyl fingerprint spectra in a much shorter time in approximately 30 min [52]. It should be noted that an international effort of standardization to make the 2D NMR method a highly confident routine way to assess HOS of mAb is underway [63,64,65,66].

## 5. The ^15^N-Detected CRINEPT for the HOS Analysis of a mAb under Formulated Storage Conditions

As mentioned above, ^1^H-detected multidimensional NMR experiments allow site-specific observation of mAbs at high temperature. Measuring at higher temperature, however, possibly induces the deterioration and aggregation of the biologics, due to structural and chemical heterogeneity of mAbs that might not exist at lower temperatures. In addition, the aforementioned experiments yield almost no signal at 4 °C [67] (Figure 2; bottom). This is simply because a slower tumbling of mAbs at lower temperatures enhances the transverse relaxation of NMR coherences. At typical storage temperature (4 °C), the rotational correlation time (*τ*_c_) of proteins is estimated to be two-fold greater at than at 32 °C. This means that a mAb behaves like a 300 kDa protein at 4 °C. Therefore, a strategy to measure the mAb NMR signals at a wider range of temperatures, including low storage temperature, and under various formulation conditions is required.

**Figure 3:**
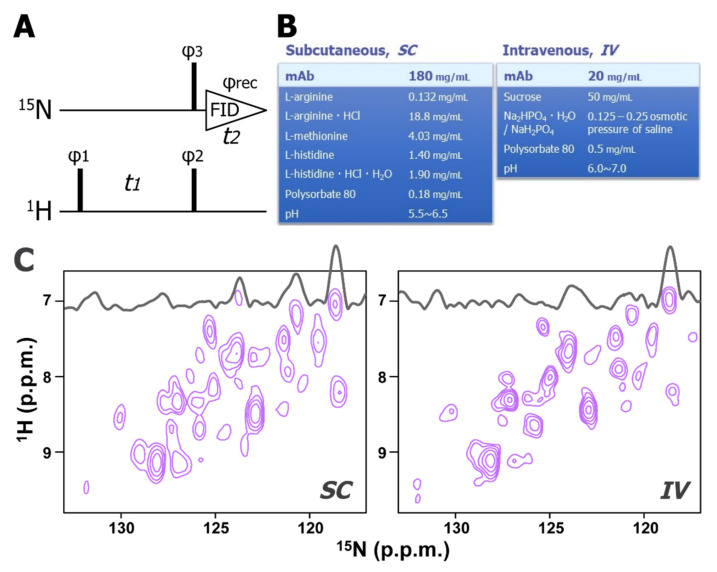
Two-dimensional ^15^N-detected CRINEPT spectra of mAb in formulated storage condition. (**A**) The pulse sequence of the ^15^N-detected CRINEPT experiment. Narrow bars indicate π/2 hard pulses. The phase cycles employed were φ1 = (x−x), φ2 = y, φ3 = (x−x−x−x), and φrec = (x−x−x−x). Phase-sensitive acquisition in the indirect ^1^H dimension is achieved by incrementing the phase φ1 in a STATES-TPPI manner [71]. (**B**) Examples of formulation condition for subcutaneous injection (SC; left), and intravenous injection (IV; right) (**C**) The ^15^N-detected CRINEPT spectra of the mAb in the SC (left) and IV (right) formulations. These spectra were acquired at 4 °C. The figure is reproduced from [67].

We recently developed a novel ^15^N-direct detection NMR experiment, ^15^N-detected CRINEPT, which allows high-sensitive and high-resolution measurements of non-deuterated high molecular weight proteins (Figure 3A). The principles of the coherence transfer in the ^15^N-detected CRINEPT is the same as in the ^1^H-detected CRINEPT [68]; however, for high-sensitive detection, several tricks are introduced, such as the concatenation of the ^1^H-chemical shift evolution and the ^1^H to ^15^N coherence transfer periods, and the ^15^N-direct detection of antiphase coherence. For a 150 kDa protein at 4 °C, the sensitivity of the ^15^N-detected CRINEPT was expected to be >20-fold greater than that of the previously reported insensitive nuclei enhanced by polarization transfer (INEPT)-based experiment [69,70]. Since the ^15^N transverse relaxation rate is less sensitive to the presence of remote protons, the direct ^15^N-detection scheme employed in the experiment does not require protein deuteration. This feature is essential for evaluating the HOS of biologics that are mostly obtained from mammalian cells. This technique was applied to an intact mAb at a wide range of temperatures (4–32 °C) and formulation conditions, including those used for long-term storage, for subcutaneous injection (SC), and intravenous injection (IV). These formulation conditions contain amino acids, surfactants, and sugars (Figure 3B), however, any of these additives do not yield unwanted signals in ^15^N-detected CRINEPT spectrum. Using the ^15^N-detected CRINEPT technique, a large fraction of ^1^H-^15^N correlation signals of mAbs was successfully observed under formulated storage conditions; where conventional ^1^H- and ^15^N-detected experiments do not work (Figure 3C).

**Figure 4:**
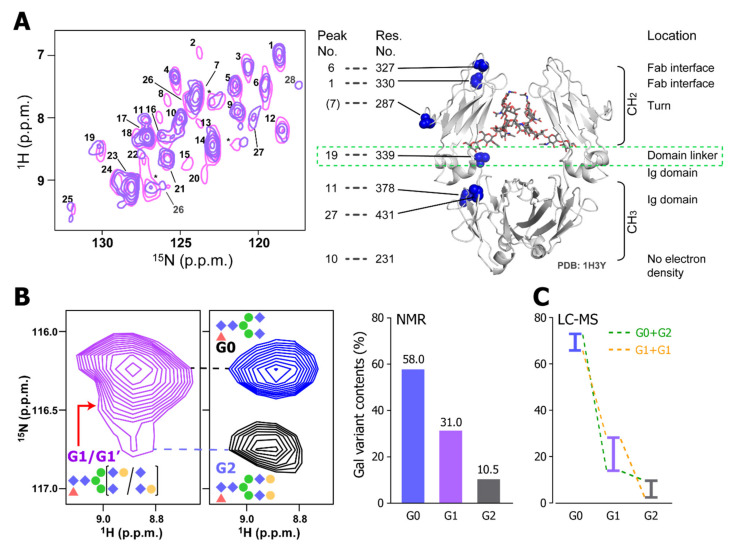
The HOS analysis of a mAb using a ^15^N-detected CRINEPT experiment. (**A**) Overlaid ^15^N-detected CRINEPT spectra of [^15^N-Ala] mAb in SC (pink) and IV (blue) formulations. The assignments are based on the chemical shifts of the Ala resonances of the Fc fragment. as previously reported [51]. The structure of the Fc fragment (1H3Y) [72] is shown along with the positions of Ala residues. Signals from ^15^N TROSY components are labeled with numbers. (**B**) The heterogeneity of the galactosylation states analyzed by the ^15^N-detected CRINEPT experiment. Left: the ^1^H–^15^N resonance of Lys-248 in the ^15^N-detected CRINEPT spectrum of the intact mAb is shown along with those from the ΔGal (blue) and Gal (black) samples, which are enzymatically galactosidated and galactosylated, respectively. Right: ratios of different galactosylation states, estimated from the deconvolution of the Lys-248 resonance in the intact mAb. (**C**) Ratios of different galactosylation states by LC–MS analysis. Reflecting that the analysis cannot discriminate G0_G2 and G1_G1 states, each bar represents the upper and lower limits of the indicated population. The figure is reproduced from [67].

The spectral patterns of mAbs in different formulation conditions were similar but not identical. This indicates that the mAb might take slightly different conformations in these formulations. It demonstrates that the ^15^N-detected CRINEPT is suitable to analyze the effect of additives to the HOS of mAbs. Especially, in SC and IV conditions, Ala-339 in the CH_2_ and CH_3_ domain linker showed different chemical shifts, indicating that the site is sensitive to the small difference in the formulation (Figure 4A). In addition, by using the ^15^N-detected CRINEPT spectra, the heterogeneity of galactosyl modifications at the tip of the N-linked sugar chain was quantitatively characterized in the intact mAb (Figure 4B). The resonance originating from Lys-248 seemed to consist of multiple components that were dispersed in the ^15^N dimension, reflecting the structural variation of mAb, depending on polymorphic glycosylation states. The glycosylation states quantified by NMR agreed with those estimated by liquid chromatography–mass spectrometry (Figure 4C). Therefore, the ^15^N-detected CRINEPT is suitable for a non-invasive HOS evaluation of the biologics.

## 6. Future Perspectives

As discussed above, the structural characterization of mAbs by NMR is powerful in obtaining HOS information of mAb as well as their stability in formulation conditions. The multifaced information from NMR will further accelerate the research and development of future mAb therapeutics. In particular, the ^15^N-detected CRINEPT experiment will be useful in such analysis as it can be utilized in a wide range of conditions, including low-temperature storage conditions. In addition, next-generation mAbs such as antibody-drug conjugates and bispecific antibodies are developing [73,74]. Upon the chemical conjugation, a mAb can change its local structure, stability, and oligomerization states. Such information could readily be detected by multidimensional NMR experiments. In addition, the relative orientation of Fab would be important to define the arrangement of antigens to exert specific functions. The combination of the ^15^N-detected CRINEPT experiment with a residual dipolar coupling experiment, for example, might provide information that can define the relative orientation of the Fab in solution. Stable isotope labeling is practically useful; however, considering that the strict regulation of pharmaceutical production, utilization of an isotope-labeled amino acid in the production stage is not trivial. It should be also noted that the versatility of NMR would allow observing and analyzing IgG in its functional site, such as in serum [75]. Nevertheless, the efforts to further expand the merit of NMR in the HOS analysis of mAbs would be important at various stages in research, development, and clinical applications.

## Abbreviations

NMR	Nuclear magnetic resonance
HOS	High ordered structures
mAb	Monoclonal antibody
PTM	Posttranslational modification
ADCC	Antibody-dependent cell-mediated cytotoxicity
ADCP	Antibody-dependent cellular phagocytosis
CDC	Complement-dependent cytotoxicity
C1q	Complement 1q
PROFILE	PROtein FIngerprint by Line shape Enhancement
STD	Saturation transfer difference
DEST	Dark-state exchange saturation transfer
2D	Two-dimensional
TROSY	Transverse relaxation optimized spectroscopy
CRINEPT	Cross-correlated relaxation-enhanced polarization transfer
SOFAST	Selective optimized flip angle short transient
HMQC	Heteronuclear multiple quantum correlation
INEPT	Insensitive nuclei enhanced by polarization transfer
NUS	Non-uniform sampling
SC	Subcutaneous
IV	Intravenous
